# Hydroxychloroquine reduces heart rate by modulating the hyperpolarization-activated current *I*_f_: Novel electrophysiological insights and therapeutic potential

**DOI:** 10.1016/j.hrthm.2015.05.027

**Published:** 2015-10

**Authors:** Rebecca A. Capel, Neil Herring, Manish Kalla, Arash Yavari, Gary R. Mirams, Gillian Douglas, Gil Bub, Keith Channon, David J. Paterson, Derek A. Terrar, Rebecca-Ann B. Burton

**Affiliations:** * Department of Pharmacology, University of Oxford, Oxford, United Kingdom; † Department of Physiology, Anatomy and Genetics, University of Oxford, Oxford, United Kingdom; ‡ Division of Cardiovascular Medicine, Radcliffe Department of Medicine, University of Oxford, John Radcliffe Hospital, Oxford, United Kingdom; § Department of Computer Science, University of Oxford, Oxford, United Kingdom

**Keywords:** ANOVA, analysis of variance, AP, action potential, HCQ, hydroxychloroquine, HR, heart rate, *I*_CaL_, L-type calcium ion current, *I*_f_, funny current, *I*_Kr_, rapid delayed rectifier potassium current, LV, left ventricle, PSS, physiological saline solution, SAN, sinoatrial node, SBA, specific bradycardic agent, SDD, spontaneous diastolic depolarization, V50, voltage of half-activation, Hydroxychloroquine, Electrophysiology, Heart failure, Arrhythmia, Pacemaker, Heart rate, Ion channels, Funny current, *I*_f_

## Abstract

**Background:**

Bradycardic agents are of interest for the treatment of ischemic heart disease and heart failure, as heart rate is an important determinant of myocardial oxygen consumption.

**Objectives:**

The purpose of this study was to investigate the propensity of hydroxychloroquine (HCQ) to cause bradycardia.

**Methods:**

We assessed the effects of HCQ on (1) cardiac beating rate in vitro (mice); (2) the “funny” current (*I*_f_) in isolated guinea pig sinoatrial node (SAN) myocytes (1, 3, 10 µM); (3) heart rate and blood pressure in vivo by acute bolus injection (rat, dose range 1–30 mg/kg), (4) blood pressure and ventricular function during feeding (mouse, 100 mg/kg/d for 2 wk, tail cuff plethysmography, anesthetized echocardiography).

**Results:**

In mouse atria, spontaneous beating rate was significantly (*P* < .05) reduced (by 9% ± 3% and 15% ± 2% at 3 and 10 µM HCQ, n = 7). In guinea pig isolated SAN cells, HCQ conferred a significant reduction in spontaneous action potential firing rate (17% ± 6%, 1 μM dose) and a dose-dependent reduction in *I*_f_ (13% ± 3% at 1 µM; 19% ± 2% at 3 µM). Effects were also observed on L-type calcium ion current (*I*_CaL_) *(*12% ± 4% reduction) and rapid delayed rectifier potassium current (*I*_Kr_) (35% ± 4%) at 3 µM. Intravenous HCQ decreased heart rate in anesthetized rats (14.3% ± 1.1% at 15mg/kg; n = 6) without significantly reducing mean arterial blood pressure. In vivo feeding studies in mice showed no significant change in systolic blood pressure nor left ventricular function.

**Conclusions:**

We have shown that HCQ acts as a bradycardic agent in SAN cells, in atrial preparations, and in vivo. HCQ slows the rate of spontaneous action potential firing in the SAN through multichannel inhibition, including that of *I*_f_.

## Introduction

Laurent et al^1^ described heart rate (HR) as one of the major determinants of myocardial oxygen consumption. Resting HR is an important predictor of cardiac mortality^2^ and has emerged as a therapeutic target. Accordingly, agents that reduce HR without affecting ventricular contractility are of major clinical interest for the treatment of ischemic heart disease and heart failure.^3^ HR reduction can be achieved with β-adrenoceptor antagonists or rate-limiting calcium channel blockers; however, these agents may exert concomitant negative inotropic and hypotensive effects,^4^ potentially exacerbating myocardial ischemia.

In 1987, Kobinger and Lillie^5^ described a novel class of substances known as specific bradycardic agents (SBAs), which induce sinus bradycardia at a concentration without detrimental hemodynamic effects. SBAs have been shown to reduce cardiac oxygen demand by increasing the diastolic period, which induces an elevation of subendocardial blood flow.^6^ The only known SBA on the market, ivabradine (S16257), is an agent that blocks the “funny” current (*I*_f_) and that acts to slow sinus node action potential (AP) firing directly in pacemaking cells.^7^

Interestingly, early work in the late 1950s^8^ explored the possibility of using chloroquine or hydroxychloroquine (HCQ) in the treatment of atrial fibrillation (without much understanding of the mode of action), with both drugs demonstrating possible efficacy (although without placebo-group comparison). Since then, there have been case reports of a potential bradycardic action of hydroxychloroquine (HCQ),^3^, ^9^, ^10^ which have led us to investigate whether this effect can be observed in cardiac preparations. HCQ was synthesized in 1950 by Surrey and Hammer.^11^ Dennis et al^12^ showed HCQ to be an immunomodulating agent. It is now widely prescribed for its antimalarial and antirheumatic effects.^13^ Here we describe novel SAN-inhibiting properties of HCQ in isolated cardiac preparations and in vivo anesthetized animals. We also investigated the safety of HCQ by in vivo oral feeding on blood pressure and cardiac contractility. The effects of HCQ are consistent across species, from single cells to multicellular preparations. Taken together, our results support the hypothesis that HCQ may be a viable, low-cost pharmacologic agent to reduce HR.

## Methods

Animal experiments are described in accordance with Animal Research: Reporting of In Vivo Experiments (ARRIVE) guidelines^14^ and conform to the Animals (Scientific Procedures) Act 1986 (UK).^15^ Procedures were performed under British Home Office license PPL 30/3080.

### Isolated cardiac preparations

An expanded Materials and methods section is available in the Supplementary Material online.

#### Mouse atrial preparations

Hearts were rapidly excised from male CD-1 mice (7–9 wk old) and washed in warm, oxygenated physiological saline solution (PSS). The preparation was hung in a 37°C organ bath filled with PSS. Beating rate was calculated in real-time from the upstroke of the tension signal. Drugs were added cumulatively, directly to the organ bath.

#### Guinea pig SAN cell isolation

Hearts were excised from male guinea pigs (350– 500 g) and rinsed in modified Tyrode solution. SAN myocytes were then isolated enzymatically.

### Single-cell electrophysiology

Perforated patch clamp recordings were carried out, using amphotericin (250 µg/mL) to achieve perforation.

#### AP recordings

APs were recorded from single guinea pig SAN cells under current clamp conditions.

#### Voltage clamp recordings

Full current–voltage relations for *I*_*f*_ and L-type calcium ion current (*I*_CaL_) as well as rapid delayed rectifier potassium current (*I*_*Kr*_) measurements were taken at 0 minutes and 5 minutes of exposure to HCQ. Please refer to the supplementary material online.

### Rat in vivo, invasive hemodynamic studies

HR and arterial blood pressure were measured under general anesthesia in male Sprague Dawley rats (300–350 g), via cannulation of the left carotid artery.

### Mice in vivo, noninvasive blood pressure by tail cuff plethysmography and cardiac contractility studies by echocardiography

Automated noninvasive tail cuff plethysmography (Visitech 2000; Visitech Systems, Apex, NC) was used to determine systolic blood pressure in response to HCQ in drinking water, which was compared with that of a control group. Echocardiography was performed at the end of the study in both groups.

### Statistics

#### In vitro statistics

For in vitro statistics, data are presented as means plus or minus standard error (SEM) and analyzed using 1- or 2-way analysis of variance (ANOVA) and with repeated measures where appropriate. Post hoc tests used the Dunnett correction. *P* values < .05 were considered statistically significant.

#### In vivo statistics

For in vivo statistics, data are presented as means +/- SEM and all data passed a normality test. Within group comparison are made using a 1-way ANOVA, with post hoc analysis to determine significance (Newman–Keuls, *P* < .05). Where in vivo data from 2 groups are compared, these data were analyzed with an unpaired 2-tailed *t* test.

## Results

### Bradycardic effects of HCQ on isolated mouse atrial preparation

**Figure 1:**
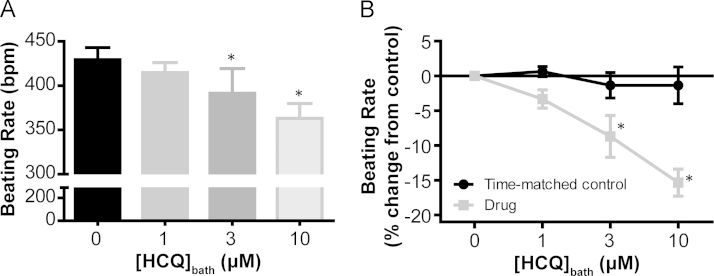
Dose-dependent effect of hydroxychloroquine on spontaneous beating rate in mouse atrial preparations. **A:** A bar graph showing the effect of cumulative doses of hydroxychloroquine (HCQ) on the sinoatrial node (SAN) beating rate in spontaneously beating preparations (36°C ± 1°C). HCQ elicited a reduction in beating rate (*P* < .05, 1-way analysis of variance ANOVA), which was significantly different from that of the control group at 3 μM (9% ± 3% reduction from the control rate, *P* < .05) and was further reduced at 10 μM (15% ± 2% reduction from the control rate, *P* < .05). For all concentrations, n = 7. *Significant difference from 0 µM. **B:** A line graph to compare the percentage change in atrial beating rate during cumulative HCQ doses with that in time-matched controls. There was no significant effect of time on beating rate in control preparations (*P* > .05, 2-way ANOVA, n = 6 control and n = 7 HCQ). *Significant difference between drug and time-matched controls by post hoc testing.

The application of cumulative doses of HCQ to spontaneously beating mouse atrial preparations revealed a dose-dependent reduction in beating rate (*P* < .05) that was significant at a dose of 3 µM (9% ± 3% slower than control rate, *P* < .05) and further enhanced at 10 µM (15% ± 2% slower than control rate, *P* < .05). Figure 1 demonstrates the beating rate in beats per minute and rate change in relation to control for all concentrations applied. No rate change was seen during time-matched control experiments in which the method was repeated without the addition of HCQ (1.4% ± 2.6% decrease from control in 2 h, *P* > .05, n = 6). Starting rates for these 2 data sets, time-match control and HCQ application, were not significantly different (*P* = .72).

To confirm that the effects of HCQ on rate reduction were likely to arise from the modulation of *I*_f_ without the significant contribution of other mechanisms, we performed a sequential accumulation protocol similar to those performed by Verrier et al.^16^ Near-maximal inhibition of *I*_f_ was achieved using 1 µM ZD7288 (*I*_f_ inhibitor), followed by cumulative doses of HCQ (Supplementary Figure 1). Applied alone, 1 µM ZD7288 caused a significant fall in spontaneous beating rate (-38% ± 5% from that with PSS, *P* < .05, n = 7). On the background of this *I*_f_ inhibition, the cumulative additions of 3 (n = 5) and 10 µM (n = 6) HCQ did not confer any additional rate change (0% ± 2% and 5% ± 5%, respectively; *P* = .89).

### Effects of HCQ on spontaneous firing frequency in isolated guinea pig SAN cells

**Figure 2:**
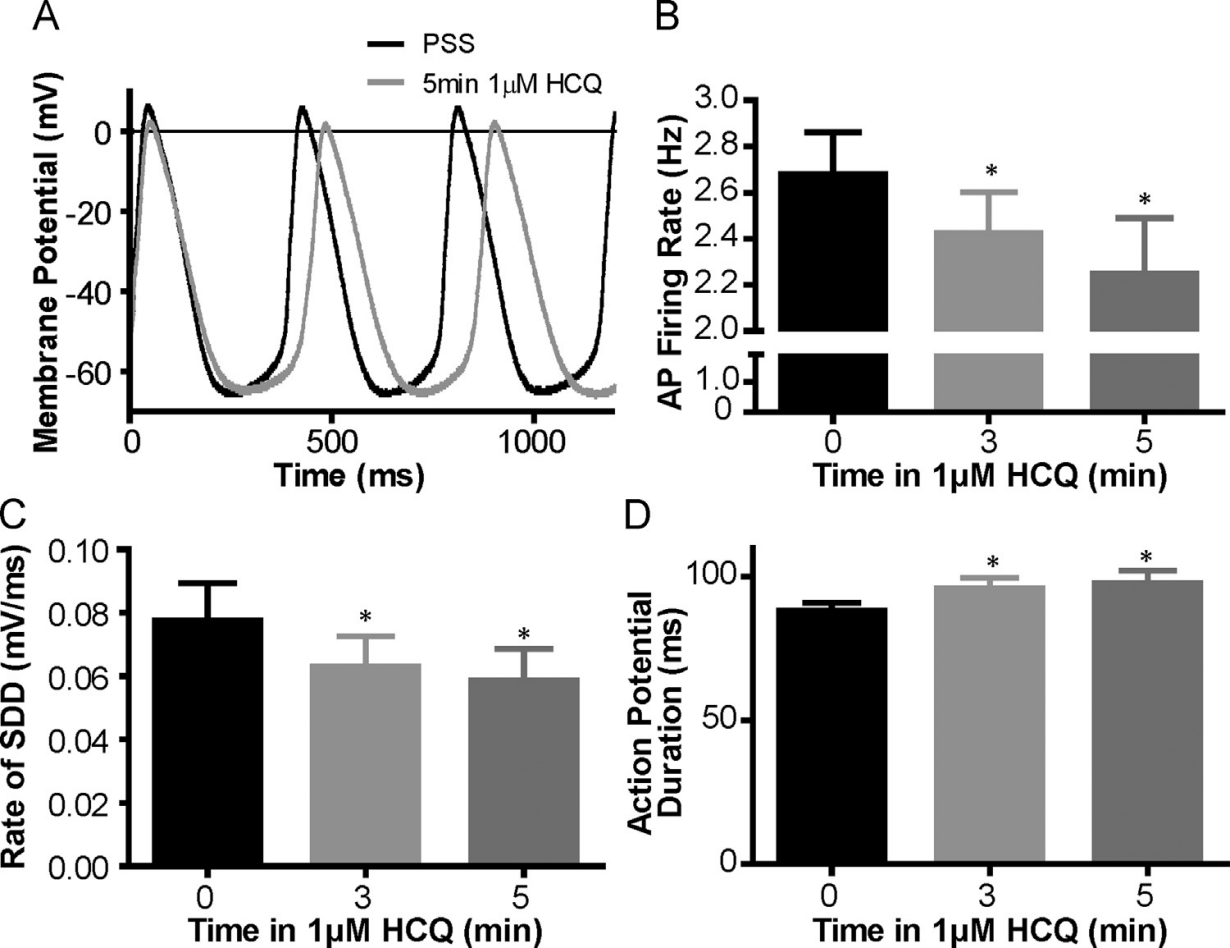
**A:** Representative traces to show spontaneous action potentials recorded from the same, isolated sinoatrial node (SAN) myocyte under perforated patch conditions before and after 1 μM hydroxychloroquine (HCQ) (35°C ± 2°C). **B:** HCQ reduces the action potential firing rate in isolated guinea pig SAN cells. A summary bar graph to show absolute action potential firing rate in Hertz before and during 1 μM HCQ application (n = 6). The action potential firing rate was significantly slowed by superfusion of 1 μM HCQ (*P* < .05, 1-way analysis of variance), with slowing significant by 3 min of exposure (10% ± 3% reduction, *P* < .05) and further reduced at 5 min (17% ± 6% reduction, *P* < .05). *Significant difference from control. The same 6 cells were further analyzed to determine action potential characteristics: 1 μM HCQ **C:** significantly reduced the rate of spontaneous diastolic depolarization (SDD) and **D:** significantly lengthened the action potential duration. *Significant effect by 1-way analysis of variance with the Dunnett correction.

Historically, mechanistic studies of SAN have focused on the guinea pig and rabbit as experimental models (in terms of AP shape and length in comparison with those of humans).^7^ Therefore, the effect of HCQ on spontaneous frequency was also tested in isolated SAN myocytes from the guinea pig. A representative trace to show SAN APs recorded before and after HCQ exposure is provided in Figure 2A. Consistent with data collected from multicellular preparations, these results show that HCQ induced a significant reduction in the rate of spontaneous AP generation. This finding amounted to a -17% ± 6% change in 5 minutes at a dose of 1 µM (*P* < .05, n = 6, Figure 2B). In the absence of drug intervention, cells that were patched but not treated with HCQ exhibited no significant change in rate in 5 minutes (0.9% ± 2.0%, *P* = .83, n = 8, data not shown). Further analysis of AP recorded during HCQ experiments revealed that slowing was accompanied by a 25% ± 3% reduction in the slope of spontaneous diastolic depolarization (*P* < .05, n = 6, Figure 2C) and an 11% ± 3% lengthening in AP duration (*P* < .05, n = 6, Figure 2D). AP amplitude, maximal upstroke velocity, and maximum diastolic potential were not significantly altered during a 5-minute, 1-µM HCQ application (see Supplementary Table 1 for summary values).

### Effects of HCQ on *I*_f_, *I*_*Kr*_*, and I*_*CaL*_ in isolated guinea pig SAN cells

The striking reduction in the slope of diastolic depolarization observed in single-cell experiments (Figure 2C) led us to investigate the effects of HCQ on *I*_f_ as a first possible mechanism by which observed rate-slowing may occur. *I*_f_ recordings were made, using a voltage clamp, of healthy guinea pig SAN myocytes from a holding potential of -40 mV to a range of hyperpolarizing voltages, in 10-mV increments, from -50 to -120 mV.

**Figure 3:**
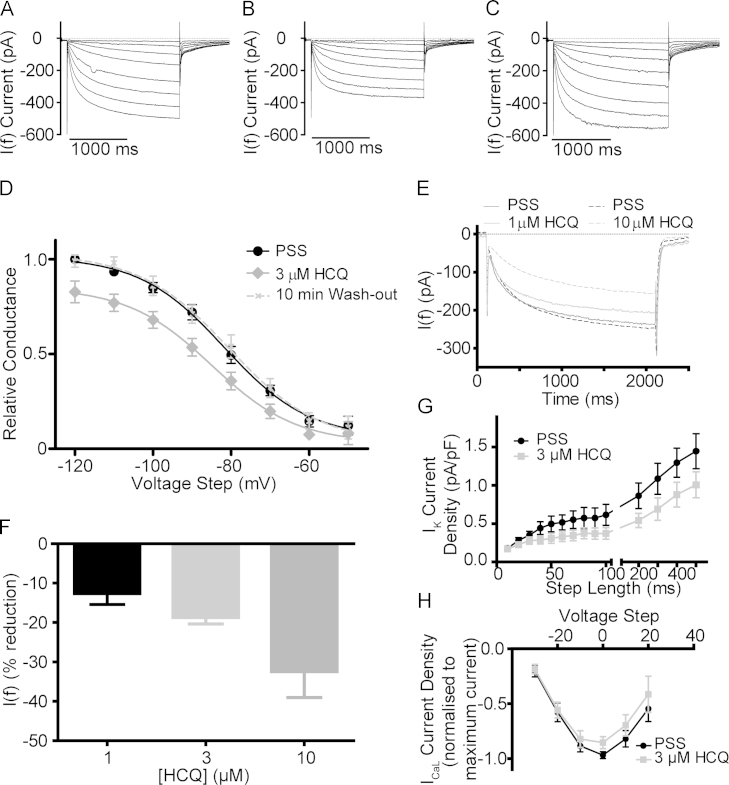
Representative traces showing current–voltage relations under **A:** control conditions (in physiological saline solution [PSS]), **B:** after a 5-min exposure to 3 µM hydroxychloroquine, and **C:** after 10 min of wash-out by return to PSS. **D:** Conductance curves (n = 4 cells) with conductance plotted relative to maximal activation of *I*_f_ under control conditions (PSS). Maximal conductance was significantly reduced over 5 min (to 85% ± 6% of control), whereas voltage of half-activation (V50) and slope of conductance were unchanged. **E:** Representative curves illustrating the effect of hydroxychloroquine at 1 and 10 µM on voltage steps to -100 mV. Data collected using repeated voltage steps to -100 mV at a rate of 0.05 Hz over 5-min HCQ exposure. **F:** Change in *I*_f_ at the -100 mV step across 3 concentrations (5-min exposure). Significant effect of concentration (*P* < .05) by analysis of variance (ANOVA), n = 7 for 1 µM, n = 5 for 3 µM, and n = 4 for 10 µM of HCQ. **G:** Potassium current density under control and 3 µM HCQ conditions (significant effect, *P* < .05, of “drug” by 2-way ANOVA, n = 5) with reduction in maximal *I*_Kr_ (average of 80-, 90-, and 100-ms step lengths) of 35% ± 4%. **H:***I*_CaL_ current–voltage relations with current normalized to maximal current density step show a significant effect of 3 µM HCQ on *I*_CaL_ (P < .05, 2-way ANOVA, n = 6) with reduction in the maximal current (0-mV step) of 12% ± 4%.

Figure 3 provides representative traces of *I*_f_ measured across the full range of voltage steps under control conditions, after 5 minutes of HCQ superfusion (3 µM), and at 10 minutes after a return to PSS (Figure 3, Figure 3, Figure 3 respectively). The superfusion of 3 µM HCQ resulted in a significant reduction in the size of *I*_f_ within 5 minutes, for instance by 20% ± 4% at the voltage step of -100 mV (n = 4).

The maximal conductance of *I*_f_ was significantly reduced by 3 µM HCQ (to 85% ± 6% of the maximal value recorded in PSS, n = 4, Figure 3D). This reduction was not accompanied by an effect on the voltage of half-activation (-81.8 ± 1.7 mV in the control group, -85.0 ± 2.6 mV in the HCQ group) nor the slope of activation (10.86 ± 1.85 vs 10.83 ± 2.93) as determined by the fitting of a Boltzmann sigmoidal function.

As shown by the example provided in Figure 3C and the conductance curve in Figure 3D, a return to PSS resulted in a complete recovery of *I*_f_ by 10 minutes (n = 4).

Interestingly, and in contrast to data reported during exposure to ivabradine,^17^ the average percentage reduction exhibited on a step to a potential likely to be encountered during physiological pacemaker cell firing (-50 or -60 mV) was greater than the reduction exhibited at a more extreme hyperpolarization (-70 mV or beyond). Taking an average of the change in raw current at the -50 and -60 mV hyperpolarization steps, the reduction in raw current was 47% ± 9% (n = 8, where n is change at 1 voltage step in 1 cell), whereas at potentials from -70 to -120 mV, the reduction in raw current was 24% ± 3% (n = 24). The “physiological” group experienced a significantly higher percentage reduction (*P* < .05 by Student *t* test).

In multicellular preparations, the effect of HCQ on spontaneous beating rate was observed to be dose-dependent. If the observed inhibition of *I*_f_ is responsible for these rate changes, it should follow that the effect of HCQ on this current is also dose-dependent. We investigated the effect of HCQ on *I*_f_ across 3 doses: 1, 3, and 10 µM using repeated 2-second steps to -100 mV delivered at 20-second intervals. Representative traces are shown in Figure 3E. At all doses tested, we observed a significant reduction in the amplitude of *I*_f_ measured by these step hyperpolarizations. This inhibition was dose-dependent (*P* < .05, 1-way ANOVA). The maximum effect was 13% ± 3% at a dose of 1 µM (n = 7), 19% ± 2% at 3 µM, (n = 5), and 32% ± 7% at 10 µM (n = 4). These data are summarized in Figure 3F.

We also performed a simulation using the mathematical SAN electrophysiology model of Dokos et al^18^ to reproduce the physiological and pharmacologic HR modulation (details and a link to open source code in Supplementary Material). This results in a 9.7% prolongation in AP duration at 50% of repolarization at full block, with a reduction in AP firing rate from 2.6 to 2.3 Hz (from 156 to 138 beats per minute), as shown in Supplementary Figure 2. The changes in firing rate (Figure 2, Figure 2) and AP duration (Figure 2D) are reproduced relatively well by the simulation of a block of *I*_f_ solely.

We studied the effects of 3 µM of HCQ on *I*_Kr_ and *I*_CaL_ and observed a significant effect on these currents. A dose of 3 µM HCQ caused a significant reduction in the amplitude of potassium tail currents ( *P* < .05, effect of “drug,” 2-way ANOVA, n = 5, Figure 3G) with a 35% ± 4% reduction in maximal *I*_Kr_ (average of current at 80-, 90-, and 100-ms step durations). Representative traces to show potassium tail currents before and after 5 minutes of exposure to 3 µM HCQ are presented in Supplementary Figures 3A and 3B. The effects of 3µM HCQ on *I*_Kr_ and estimated *I*_*Ks*_ are presented in Supplementary Figures 3C and 3D. *I*_CaL_ current density was also inhibited by 3 µM HCQ over 5 minutes (*P* < .05, effect of ‘drug’, 2-way ANOVA, n = 6, Figure 3H) with the maximal current (step to 0 mV) reduced by 12% ± 4% (Supplementary Figure 3E shows representative traces).

### Chronotropic effects of hydroxychloroquine on anesthetized adult rats

**Figure 4:**
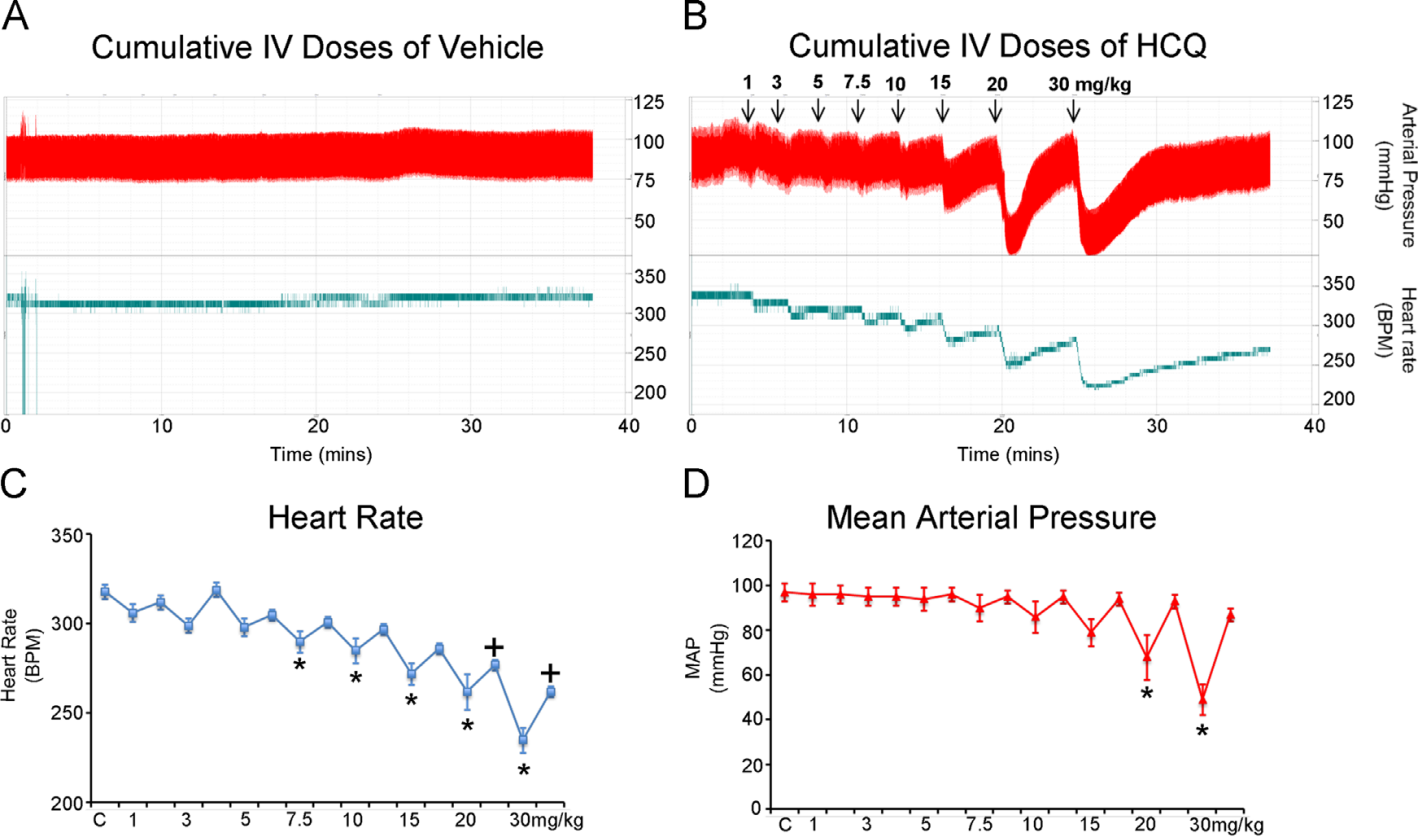
Acute in vivo experiments; anesthetized (2% isoflurane) adult Sprague Dawley rat (male), heart rate and blood pressure responses (measured directly via left carotid cannula) to cumulative intravenous doses of **A:** vehicle and **B:** hydroxychloroquine via left carotid artery. **C:** Heart rate responses and **D:** Mean arterial pressure responses. *P < .05 control (C) vs transient drop (ANOVA/Bonferroni); +*P* < .05 control (C) vs equilibrated response (ANOVA/Bonferroni).

As can be seen in the raw data trace in Figure 4, intravenous administration of HCQ produces a transient drop in HR and blood pressure, which is then compensated for over the subsequent minutes. A significant transient reduction in HR is observed at cumulative doses of 7.5 mg/kg and above (Figure 4C). At 20 mg/kg and above, HR remains significantly below the baseline rate even during the compensatory period. In contrast, mean arterial pressure is compensated for at all doses used and the transient drop in pressure is significant only at doses of 20 mg/kg and above (Figure 4D). The percentage transient change in HR at 15 mg/kg (the highest dose with no significant change in mean arterial blood pressure) is 14.3% ± 1.1%.

### Efficacy studies on blood pressure and contractile function in adult mice

**Figure 5:**
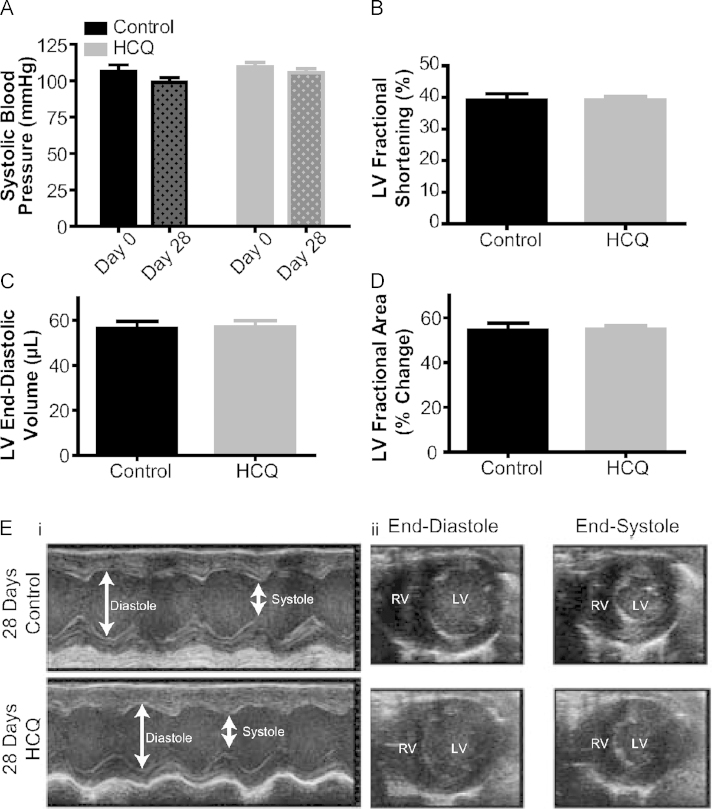
Effects of oral hydroxychloroquine on **A:** systolic blood pressure by tail cuff plethysmography (n = 9/group, dose 100 mg/kg in drinking water, 28 d). Echocardiography was performed under anesthesia with a 30-MHz linear array transducer. Assessment of **C and D:** contractile function and left ventricular end-diastolic chamber size by echocardiography at the end of the feeding study. **E: i:** Example M-mode parasternal short-axis at midventricular level in both groups (no significant difference), Arrows, End-diastolic dimension (EDd) and end-systolic dimension (ESd) of left ventricle; **ii:** Example parasternal short-axis view showing end diastole and end systole in both groups, showing no significant effect on ventricular end-diastolic volume, end-systolic volume, or left ventricular mass in comparison with that of control mice.

In view of the transient reduction in blood pressure seen on intravenous administration of HCQ, we conducted a long-term feeding study with blood pressure measurements and assessment of contractile function by echocardiography. As shown in Figure 5A, administration of HCQ (100 mg/kg) in the drinking water did not alter blood pressure from the baseline value in the treatment group (HCQ baseline 109.7 ± 2.89 mm Hg vs end of study 110.7% ± 1.91 mm Hg, *P* = not significant, n = 9 per group). All animals were weighed daily to ascertain whether they were consuming the drug, and no significant weight loss was observed in control or drug groups (control start/end weight 28.08 ± 0.29 g/31.56 ± 0.63 g, HCQ start/end weight 27.72 ± 0.36 g/29.99 ± 0.58 g). Transthoracic echocardiograms of mice treated with HCQ in drinking water in comparison with those of control mice given vehicle solution (n = 9 per group) revealed no significant effect of HCQ on left ventricle (LV) contractility, as judged by fractional shortening (HCQ group 39.1% ± 1.2%, control group 39.1% ± 2.0%), ejection fraction (HCQ group 70.3% ± 1.4%, control group 70.0% ± 2.5%), and fractional area change in myocardial cross-sectional area (HCQ group 55.0% ± 1.5%, control group 54.4% ± 3.2%); on ventricular end-diastolic volume (HCQ group 57.1 ± 2.7 µL, control group 56.4 ± 3.1 µL); or on LV mass (HCQ group 101.7 ± 5.1 mg, control group 100.8 ± 3.9 mg) in comparison with those of control mice given vehicle solution (Figures 5B–5E).

## Discussion

Pacemaking is a basic physiological process, inherent to the cells of the SAN. The basic cellular and molecular processes underlying pacemaking are a major target of cardiac therapies.^19^ Pacemaking cells exhibit spontaneous diastolic depolarization in phase 4 of the AP, which distinguishes them from the myocytes of the working muscle. This diastolic depolarization is what allows the repeated, rhythmic, spontaneous generation of APs.^19^ In the late 1970s, a hyperpolarization-activated funny current was discovered to play a major role in pacemaking mechanism in the SAN and was named *I*_f_.^20^ Today, the only approved clinical agent to target this ion channel is ivabradine, which is prescribed in a narrow therapeutic window.^21^ The antifibrillatory effects of cholorquine on different ion channels has been extensively investigated.^22^ Here we observe a reduction in *I*_f_, *I*_Kr_*,* and *I*_CaL_. To our knowledge, this is the first preclinical evidence that the widely used agent HCQ acts on the cardiac *I*_f_, leading to a reduction in conductance without concomitant effects on the voltage of half-activation.

This finding differs from the expected effect of changes in cyclic adenosine monophosphate, and therefore of autonomic signaling, which would lead to a shift of the voltage of half-activation without modulation of the maximal achievable conductance at the most negative voltage steps.^23^ Instead, the effect of HCQ on *I*_f_ conductance curves is similar to experiments measuring *I*_f_ modulation using the specific *I*_f_ blocker ivabradine.^23^ Experiments performed in guinea pig SAN cells showed that HCQ acts mainly by reducing spontaneous diastolic depolarization, without a significant change in further AP characteristics. As with all proposed SBAs,^5^ HCQ will exert effects on pacemaking mechanisms beyond modulation of *I*_f_ as dose is increased; the results of our sequential accumulation protocol with ZD7288 are promising, suggesting that HCQ does not reduce beating rate under conditions in which complete block of *I*_f_ has already been achieved. Transient bradycardia was also observed with an in vivo cumulative dose of HCQ of 7.5 mg/kg and above, initially without any effect on blood pressure. At high doses (≥20 mg/kg), the effects on blood pressure become significant.

Although debate has continued regarding the percentage contribution of *I*_f_ to pacemaking and its relative importance in relation to the “calcium clock,”^24^ the inhibition or genetic modulation of *I*_f_ has been shown to slow HR significantly and reproducibly in all the species used in this study.

Published preclinical work that has underpinned the licensing of ivabradine for human use as an SBA has included findings from mouse,^25^ rat,^26^ guinea pig,^7^ and rabbit,^27^ among other mammalian species. Whatever the precise role of *I*_f_ for basic pacemaker function, it has an important role in the autonomic modulation of HR.^28^ Our results suggest that HCQ at 1–3 µM is targeting multiple membrane ion channels including *I*_f_. Simulation studies have shown that a putative compound that blocks multiple channels (eg, *I*_Kr_*; I*_CaL;_ sodium current, *I*_Na_) can have better antiarrhythmic effects than ones that block only *I*_Kr_.^29^ Given the emergence of cardiovascular disease as a leading cause of death in low-income countries, drug repositioning of HCQ for the treatment of arrhythmias and angina pectoris may represent a novel, cost-effective, and safe means particularly suited to resource-limited settings. In addition, HCQ could be a potential therapeutic employed in combination therapies (eg, with angiotensin-converting enzyme inhibitors, calcium channel antagonists, beta blockers, and other *I*_f_ blockers).

We observed no significant change in mean arterial pressure with lower doses of HCQ in vivo, despite a lowering of HR. At first sight, this may be inferred to represent compensatory neurohumoral activation to maintain systemic arterial pressure in the face of bradycardia. Notably in this regard, in preclinical models of selective *I*_f_ inhibitor, ivabradine reduces HR without perturbing cardiac output or blood pressure by increasing stroke volume via prolongation of diastolic filing time and potentially through effects on the extracellular matrix.^30^ Long-term ivabradine treatment in human heart failure with reduced ejection fraction has been shown to reduce effective arterial elastance and increase stroke volume with no change in systemic vascular resistance, LV contractility, or cardiac output, a finding that is consistent with enhanced ventricular-arterial coupling.^31^ As a corollary, selective HR lowering by ivabradine has been shown to reduce levels of natriuretic peptide, a marker of neurohumoral activation of the myocardium, in heart failure.^32^ Interpreted in this light, the lack of effect of HCQ on mean arterial pressure at low doses supports the hypothesis that it is acting selectively on the SAN in this dose range.

At extremely high doses, mean arterial pressure was observed to be significantly reduced, which may reflect an additional vasodilatory or negative inotropic action of HCQ. To assess the efficacy of the drug, we conducted noninvasive in vivo feeding studies in mice at a dose of 100 mg/kg (highest oral dose tolerated by the mice without compromising their oral intake) using tail cuff plethysmography over 28 days, during which no significant changes in systolic blood pressure were observed. We were unable to accurately record HR because of the limitations of this technique. Echocardiography was performed on a cohort of these animals (blinded studies) with no significant changes observed in contractile function. It is unlikely that equivalent concentrations causing hypotension from intravenous administration would ever be reached through oral dosing of HCQ in patients. Moreover, hypotension is not a noted side effect of dosing for the current uses of this compound in humans.

## Conclusions

We describe novel mechanistic insights relating to the action of HCQ in cardiac preparations. We observe interesting inhibitory effects on *I*_f_, *I*_Kr_*, and I*_CaL_. Cardiac arrhythmia is not a reported side effect of HCQ, despite its widespread use in healthy (antimalarial) and diseased (rheumatological) populations. It is possible that blockade of *I*_CaL_ could be countering some of the effect of the hERG channel block.^29^ The experiments described herein studying the funny current closely mirror those first performed to investigate the effects of known *I*_f_ blockers (eg, ZD7288, ivabradine), and suggest that further studies regarding cardiac modulation by HCQ is warranted.

**Clinical Perspectives**

Elevated heart rate (HR) is recognized as a predictor of adverse cardiac outcomes and has emerged as a treatment target in its own right. Lowering HR and thereby myocardial oxygen demand is a well-established strategy to treat angina. Results from the recent SHIFT (Systolic heart failure treatment with the *I*_f_ inhibitor) trial evaluating the effect of ivabradine on cardiovascular outcomes, symptoms, and quality of life in patients with chronic heart failure and systolic dysfunction confirm the pivotal role of HR in the pathophysiology of heart failure and support the strategy of HR reduction for the improvement of clinical outcomes.^33^ Our study identifies HCQ, a well-established agent used in the treatment of malaria and inflammatory disorders for over half a century, as having a novel specific HR-lowering effect. Using electrophysiological and drug-combination experiments across a number of mammalian species, we identify inhibition of the cardiac pacemaker’s hyperpolarization-activated current, *I*_f_, as one of the possible mechanisms underlying this rate reduction. Although HCQ has a well-studied toxicity profile,^34^ our findings highlight the need for the monitoring of HR in patients on high doses of HCQ and the need for caution with coadministration of bradycardic agents. Given the inexpensive nature and good tolerability profile of HCQ that has been established over decades of clinical experience, our findings point to a potential novel role for this old drug as a cost-effective selective HR slowing agent that may be readily tested and translatable to resource-limited settings worldwide to treat the global epidemic of cardiovascular disease.

## Disclosure

International patent application number PCT/GB2014/052109, July 2014. For further information regarding intellectual property and licensing, please contact Dr Matthew Carpenter, Isis Innovation, UK (http://matthew.carpenter@isis.ox.ac.uk).
