# Supplementary material for: Hydroxychloroquine reduces heart rate by modulating the hyperpolarization-activated current If: Novel electrophysiological insights and therapeutic potential
Source: Heart Rhythm. 2015 Oct;12(10):2186–94. doi: 10.1016/j.hrthm.2015.05.027 (PMC4689153; doi:10.1016/j.hrthm.2015.05.027)
Supplement: Supplementary file 1 — Supplementary Material [file mmc1.docx]

**Supplemental Material**

**Hydroxychloroquine reduces heart rate by modulating the hyperpolarisation-activated current ‘*I*_f_’: Novel electrophysiological insights and therapeutic potential**

Short title: Capel, Hydroxychloroquine as a rate reducing agent

Rebecca Anne Capel^1^, Neil Herring^2^, Manish Kalla^2^, Arash Yavari^3^, Gary R Mirams^4^, Gillian Douglas^3^, Gil Bub^2^, Keith Channon^3^, David J Paterson^2^, Derek Terrar^1^ and Rebecca-Ann B Burton°^2^

^1^Department of Pharmacology, University of Oxford, Mansfield Road, Oxford OX1 3QT

^2^Department of Physiology, Anatomy and Genetics, University of Oxford, Parks Road, Oxford OX1 3PT

^3^Division of Cardiovascular Medicine, Radcliffe Department of Medicine, University of Oxford, John Radcliffe Hospital, Oxford OX3 9DU

^4^Department of Computer Science, University of Oxford, Oxford OX1 3QD

°Corresponding author

**Supplemental Methods:**

All experiments conformed to the Guide for the Care and Use of Laboratory Animals published by the Animals (Scientific Procedures) Act 1986 (UK) and were carried out under the ARRIVE guidelines.[1]

1. **Isolated Cardiac Preparations:**
2. Mouse Atrial Preparations

Male CD-1 mice (7-9 weeks of age) were terminated by concussion followed by cervical dislocation, the chest cavity was opened and heparinised solution (10 U/ml) applied around the heart to minimise clotting during dissection. The heart was rapidly excised and placed in warm, oxygenated physiological saline solution (PSS, in mM: NaCl 125, NaHCO_3_ 25, KCl 5.4, NaH_2_PO_4_ 1.2, MgCl_2_ 1, glucose 5.5, CaCl_2_ 1.8, pH to 7.4 with NaOH and oxygenated with 95 % O_2_/5% CO_2_), the ventricular tissue discarded and the SAN region cleared of any overlying tissue.

Loops of thin suture were tied to the lateral edges of each atrium by directly knotting around a small area of the tissue, taking care to avoid contact with the node itself. One loop was anchored to a hook, which also provided oxygenation to the organ bath, and the other tied to a tension transducer. The preparation was hung in an organ bath filled with PSS, maintained at 37ºC. Tension data were digitised using a PowerLabs bridge amplifier and recorded on Chart5 software (all from ADInstruments, UK). Beating rate was calculated in real-time from the upstroke of the tension signal using the Chart5 Ratemeter function. Spontaneously-beating atrial preparations were allowed to stabilise for 20min, solution was exchanged for fresh PSS and the preparation was again allowed to stabilise for 30min. Drugs were added directly to the organ bath by pipette and allowed to equilibrate for 30min before rate data were sampled.

1. Guinea Pig SAN Cell Isolation

Male guinea pigs (350-500g) were sacrificed by concussion followed by cervical dislocation. The heart was rapidly excised and encouraged to beat several times in heparin-containing zero-Ca^2+^ modified Tyrode solution (in mM: NaCl 136, KCl 5.4, NaHCO_3_ 12, Na+ pyruvate 1, NaH_2_PO_4_ 1, MgCl_2_ 1, EGTA 0.04, glucose 5; gassed with 95% O_2_/5% CO_2_ to maintain a pH of 7.4), heparin to prevent clotting (10 U/ml). The heart was mounted via the aorta on a constant pressure Langendorff system for retrograde perfusion, with solutions maintained at body temperature. After 2min initial wash with zero Ca^2+^ solution, perfusion was switched to re-circulation with collagenase solution (same as zero Ca^2+^ but with 27mg collagenase/50ml – type II Worthington, Worthington Biochemical Corp., Lakewood, NJ, USA – 0.1mM CaCl_2_, no heparin and no EGTA).

Following 25 mins enzymatic digestion, the right atrium, vena-cavae and pulmonary artery were dissected away from the ventricles. The right atrium was pinned in a Sylgard-coated bath, opened by anterior incision, and the SA node identified from anatomical features and tissue appearance. The SAN was dissected into thin strips (~2x5mm) and stored in a high potassium medium (in mM: KCl 70, MgCl_2_ 5, K^+^ glutamine 5, taurine 20, EGTA 0.04, succinic acid 5, KH_2_PO_4_ 20, HEPES 5, glucose 10; pH to 7.2 with KOH). Isolation of single SAN myocytes was carried out by gentle trituration.

For all single cell experiments, cells were transferred to a glass coverslip and superfused with oxygenated PSS at 34±2ºC using a gravity-fed system running at 2-3ml/min. HCQ was applied via superfusion, with an electronic switching mechanism resulting in full solution exchange over a period of less than 20s.

**Single Cell Electrophysiology Recordings**

Perforated patch clamp recordings were carried out using electrodes pulled from filamented borosilicate glass capillary tubing (GC100F, Harvard Apparatus Ltd, Kent) using a two-stage vertical puller (Narishige, Japan)[2]. Electrode resistances were in the range of 2.8-6.4MOhm when filled with patch pipette solution containing (in mM): K^+^-aspartate 110, KCL 10, NaCl 5, MgCl_2_ 5.2, HEPES 5, K_2_ATP 5, pH to 7.2 with KOH. Patch solution also contained amphotericin (250µg ml^-1^) to achieve perforation. Amphotericin was dissolved in DMSO to a stock solution of 20mg ml^-1^. This solution was made freshly on each experimental day and the final concentration achieved by diluting into the patch solution. Amphotericin remained fully dissolved by this method.

Micropipettes were mounted in a Perspex holder containing an Ag/AgCl wire and connected to a CV203BU headstage. All recordings were carried out using an AxoPatch 200B microelectrode system, digitised with a Digidata 1200 A-D converter, and recorded using PClamp7 software at a sampling rate of 2 kHz.

Micropipettes were controlled using an electronic micromanipulator (Burleigh, USA). After positioning close to the cell of interest, the tip potential was manually compensated. Gigaseals were formed by manual suction and the cell left for up to 15 min to allow patch perforation and good electrical access using amphotericin. Cells were used in current clamp mode for the recording of action potentials or voltage clamp mode for the recording of *I*_f_ currents.

**Action Potential Recordings**

Action potentials were recorded from single guinea pig SA node cells under current clamp conditions using the AxoPatch200B microelectrode system and rate measured in Hz using spectral analysis.

**Ion Current Recordings**

Cell voltage was clamped at -40 mV in the V-clamp configuration as a holding potential in all experiments. Full current-voltage relations for *I_f_* and *I_CaL_*, and *I_K_* measurements were taken at 0min and 5min exposure to HCQ.

I_f_ was activated by step hyperpolarizations from a holding potential of −40 mV. Hyperpolarising pulses of 2 s in length were applied at 20 s intervals and *I*_f_ measured as the maximum current minus current at the start of the voltage pulse. Full current-voltage relations were taken at 0 min and 5 min exposure to HCQ by application of successive steps to -50, -60, -70, -80, -90, -100, -110 and -120 mV. Intervening voltage pulses were applied to -100mV only. Conductance values were calculated using the equation

Conductance = absolute current
 driving force

Where driving force is equal to the voltage step minus the reversal potential for *I*_f_. A reversal potential of -19.2 mV was used for all of these calculations, as published from previous work.[3] Conductance curves were fitted with a Boltzmann Sigmoidal function and analysed for changes in maximal conductance, voltage of half-activation and slope.

*I_CaL_* curves comprised 200ms step depolarisations in the range -30 to +30 mV in 10 mV increments at 5s intervals. *I_CaL_* current was measured as peak inward current minus current at the holding potential. *I_Kr_* and *I_Ks_* were activated with step depolarisations to +40mV for step lengths ranging from 10 to 100 ms in 10 ms increments (*I_Kr_* activation) and 100 to 500 ms in 100 ms increments (*I_Ks_* activation). *I_K_* was measured as maximum tail current amplitude minus current upon complete tail current recovery.

**Cardiac sino atrial node simulation using Dokos *et al.* (1996) model**

We performed simulations of steady electrical activity using a mammalian SAN model[4]. The block of If was implemented as a percentage reduction of the maximum conductance of both the sodium and potassium components of the If current. We then ran a simulation for 100 seconds, to allow the electrophysiological changes to settle to a steady state, before simulating for another 2 seconds to produce detailed output (steps of 0.1ms) which were analysed by finding the first minimum voltage, aligning the traces to this point (as shown in Supplementary Figure 2).

Simulations were performed using Chaste[5], which converts a CellML description of the model into C++ code to be solved using the adaptive time-stepping solver CVODE with tolerances of 10^-5^ (relative) and 10^-7^ (absolute). The code is open source, written to work with Chaste v3.3, and is available with an online tutorial walkthrough at <https://chaste.cs.ox.ac.uk/trac/wiki/PaperTutorials/HR2014>.

1. **Rat *in vivo*, invasive hemodynamic studies**

Heart rate and arterial blood pressure were measured invasively as a terminal procedure on male SD rats (300-350 g). General anesthesia was induced using 5% isoflurane (3 l/min oxygen) in an anesthetic chamber and then maintained via a facemask using 2% isoflurane with the animal on a pre-heated matt to maintain normal body temperature. The left carotid artery was cannulated with a 3F portex cannula and connected to a pressure transducer. Data were acquired (200 Hz) in real time using a Biopac M100 system connected to a Dell P4 computer using AcqKnowledge software. Heart rate was triggered from the arterial blood pressure signal. Intravenous access was gained via the left external jugular vein using a 3F cannula. After an equilibration period of at least 10 min, HCQ (dissolved in sterile normal saline for injection and warmed to body temperature) was given as intravenous boluses over 30 seconds to produce a cumulative dose response curve (1-30mg/kg). HCQ was administered in volumes of 50 – 250 µL and a control experiment showed that equivalent boluses of normal saline did not significantly alter arterial blood pressure or heart rate on their own. Once a stable response had been reached, measurements were taken as an average over 10 seconds. On completion of the protocol, animals were euthanised with intraperitoneal injection of pentobarbitone.

1. **Mice *in vivo*, non-invasive blood pressure and cardiac contractility studies**

Automated non-invasive tail cuff plethysmography (Visitech 2000, Visitech, USA) was used to determine systolic blood pressure in response to HCQ in drinking water and compared to a control group. n=9 animals per group would be 80% powered to detect an 8 mmHg difference between the two groups, or 85% powered to detect a 6 mmHg difference within each group at two time points. The mice were given either normal water or HCQ (100 mg/kg).[6] HCQ drinking water was prepared in a sterile environment by dissolving 400 mg in 500 ml of sterile drinking water (final concentration 0.8 mg/ml). An average 25 g mouse (weight at start of study) drinks approximately 3 ml/day (internal communication, Dr Gillian Douglas) therefore over a 24 hour period each mouse would receive 2.4 mg HCQ (100 mg/kg equivalent in 25 g mouse is 2.5 mg/day). Estimation of water consumption by volume is not feasible therefore we used body weight as a surrogate (see results section). Mice will maintain their weight if drinking normal amounts and sucrose (50 g/L of drinking water) was used to mask the taste of the drug in order to ensure adequate intake.

C57BL/6 mice were habituated for 5 days and then underwent measurements alternate day for 28 days. All measurements were taken in the morning at the same time of day. Twenty measurements were taken, the first 10 discarded and an average of valid recordings then taken for analysis.

Echocardiography was performed at the end of the study in both groups with a 30 MHz linear array transducer on a bench-mounted adjustable heating platform (Vevo 2100 Imaging System, Visualsonics, Toronto, Canada). After induction of anesthesia with 4% Isoflurane in 100% oxygen, anesthesia was maintained with 1.25% isoflurane in 100% oxygen, pre-warmed ultrasound coupling gel was applied to the shaved chest wall and images taken by an experienced operator blind to treatment group in the parasternal view with continual surface ECG and respiratory rate monitoring. Measurements of ventricular dimensions, including left ventricular cavity cross-sectional area at mid-papillary level in short-axis, were made off-line blind to treatment allocation according to American Society of Echocardiography guidelines using Vevo analysis software (v1.6.0).

**Statistics:**

*In vitro* Statistics: data are presented as means +/- standard error of the mean (SEM) and analysed using one- or two-way ANOVA and with repeated measures where appropriate. Dunnett’s post-hoc tests were used to compare specific groups to control. Conductance curves were fitted with a Boltzman sigmoidal function using Prism5 software and parameters compared using an F-test. In all cases p<0.05 was considered to indicate a statistically significant difference. All data sets represent the results of cells from at least 4 separate animal experiments. No repetition was made of the same protocol on the same cell or tissue, such that all experiments can be considered to be independent.

*In vivo* Statistics: data is presented as means +/- standard error of the mean and all data passed a normality test. Within group comparison are made using a one way ANOVA, with post-hoc analysis to determine significance (Neuman-Keuls, p<0.05). Where two group *in vivo* data are compared, these were analysed with an unpaired two-tailed t-test.

**Drugs:**

Hydroxychloroquine sulfate 7-Chloro-4-[4-(N-ethyl-N-b-hydroxyethylamino)-1-methylbutylamino]quinoline sulfate (Sigma-Aldrich, H0915), purchased as powder, initially dissolved in distilled water and further dilutions were carried out in PSS or saline, or dissolved in sterile normal saline for injection (*in vivo* experiments). For the feeding studies, 100mg/kg dosage schedule was employed, supplemented by 50g/L of sucrose (drug taste masking protocol, Veterinary Services, University of Oxford).

**Supplementary Table 1**

| **Action Potential Characteristic** | **Control** | **3 min 1µM HCQ** | **5 min 1µM HCQ** |
| --- | --- | --- | --- |
| Firing Rate (Hz) | 2.7±0.2 | 2.4±0.2* | 2.2±0.2* |
| Slope of SDD (mV/ms) | 0.078±0.012 | 0.063±0.010* | 0.059±0.010* |
| APD (ms) | 88.1±2.6 | 95.8±3.7* | 97.6±4.4* |
| MDP (mV) | -59.1±4.8 | -59.1±4.6 | -58.7±4.6 |
| Amplitude (mV) | 83±6 | 81±7 | 79±7 |
| AP Upstroke Velocity (mV/ms) | 8.4±3.4 | 8.1±3.6 | 7.8±3.7 |

**Supplementary Table 1:** Action potential characteristics from SAN cells recorded during 1 µM HCQ application over the course of 5 min. SDD = spontaneous diastolic depolarisation, APD = action potential duration measured as time to 50% repolarisation, MDP = maximum diastolic potential. * indicates significant difference (p<0.05) from control recording by repeated measures one-way ANOVA with Dunnett’s post-hoc correction. All data are presented as mean±SEM and n=6.

**Supplementary Figure 1**

Beat rate responses during sequential accumulation protocol of 1 µM ZD7288, a

specific *I*_f_ inhibitor, followed by cumulative doses of HCQ. 1 µM ZD7288 caused a significant fall in spontaneous beating rate (-38±5% from PSS, n=7, p<0.05). On the background of ZD7288 *I*_f_ inhibition, cumulative addition of 3 (n=5) and 10 (n=6) µM HCQ did not confer any additional rate change (0±2% and 5±5% from rate after full effect of 1 µM ZD7288 respectively, effect of HCQ p=0.89).

**Supplementary Figure 2**

Left: simulation of steady-state spontaneous transmembrane voltage using the Dokos *et al.* (1996)[4] sino atrial node model. Curves are shown for control (0% block) to 100% block in steps of 10%, the arrows summarising the effect of increasing block. Right: a summary of the changes to APD_50_ with increasing *I*_f_ block; a 9.7% prolongation in APD_50_ at 100% block is observed.

**Supplementary Figure 3**

(A and B) representative potassium tail current recordings under control conditions and after 5min 3 µM HCQ exposure, respectively. Step lengths presented are 50, 100, 200, 300, 400 and 500 ms. (C) Change in IKr represented as normalised to maximal activation. HCQ had a significant effect (p<0.05, two-way ANOVA, n=5) on *I_Kr_* current density over the course of 5min with reduction in maximal *I_Kr_* of 35±4% (100ms step duration). (D) Estimated *I_Ks_*, calculated by subtraction of maximal *I_Kr_* from total potassium current, normalised to maximum *I_Ks_* current at the 500 ms step duration. HCQ had a significant effect on estimated *I_Ks_* (p<0.05, two-way ANOVA, n=5) with maximum current (500ms step duration) reduced by 25±9%. (E) Representative *I_CaL_* recorded at the 0 mV step before and after 5min application of 3 µM HCQ.

**Supplemental References**

1. Kilkenny, C., et al., *Improving bioscience research reporting: the ARRIVE guidelines for reporting animal research.* PLoS Biol, 2010. **8**(6): p. 1000412.

2. Rigg, L., et al., *Localisation and functional significance of ryanodine receptors during beta-adrenoceptor stimulation in the guinea-pig sino-atrial node.* Cardiovasc Res, 2000. **48**(2): p. 254-64.

3. Rigg, L., et al., *Modulation of the hyperpolarization-activated current (I(f)) by calcium and calmodulin in the guinea-pig sino-atrial node.* Cardiovasc Res, 2003. **57**(2): p. 497-504.

4. Dokos, S., B. Celler, and N. Lovell, *Ion currents underlying sinoatrial node pacemaker activity: a new single cell mathematical model.* J Theor Biol, 1996. **181**(3): p. 245-72.

5. Mirams, G.R., et al., *Chaste: an open source C++ library for computational physiology and biology.* PLoS Comput Biol, 2013. **9**(3): p. 14.

6. Wang, Y., et al., *Preventive effect of Ophiopogon japonicus polysaccharides on an autoallergic mouse model for Sjogren's syndrome by regulating the Th1/Th2 cytokine imbalance.* J Ethnopharmacol, 2007. **114**(2): p. 246-53.
