# Supplementary figures and images for: Hydroxychloroquine reduces heart rate by modulating the hyperpolarization-activated current If: Novel electrophysiological insights and therapeutic potential
Source: Heart Rhythm. 2015 Oct;12(10):2186–94. doi: 10.1016/j.hrthm.2015.05.027 (PMC4689153; doi:10.1016/j.hrthm.2015.05.027)

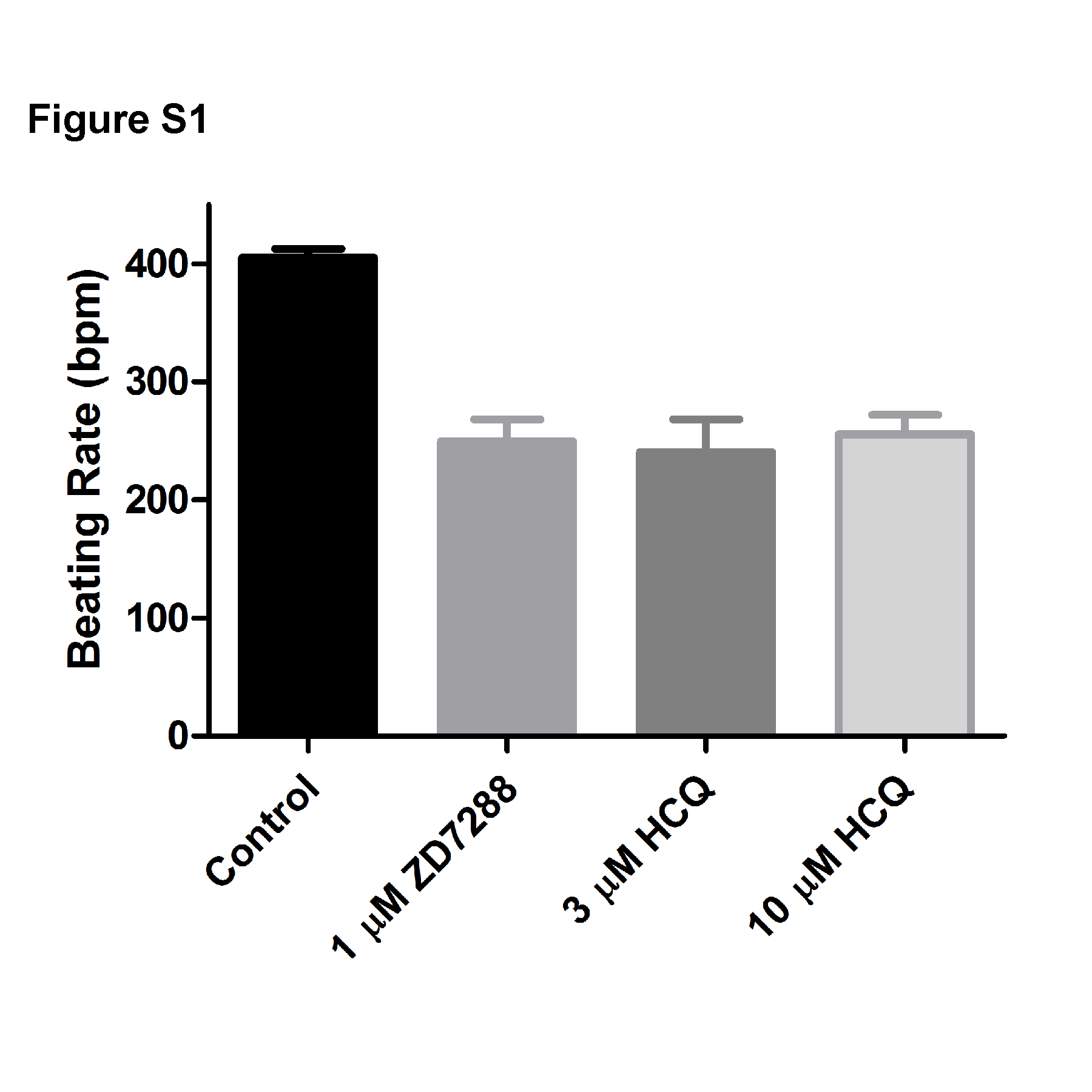

Supplement: Supplementary file 2 — Supplementary Material [file mmc2.tif]

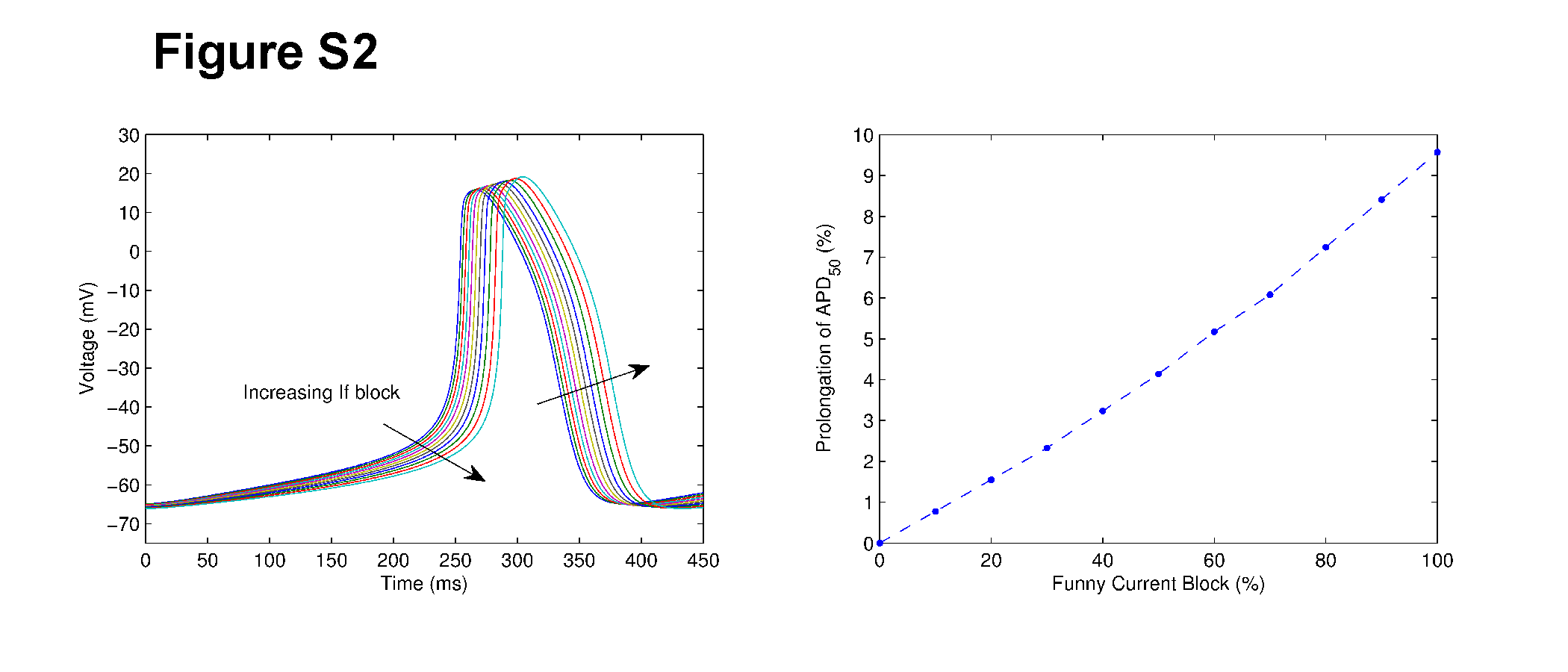

Supplement: Supplementary file 3 — Supplementary Material [file mmc3.tif]

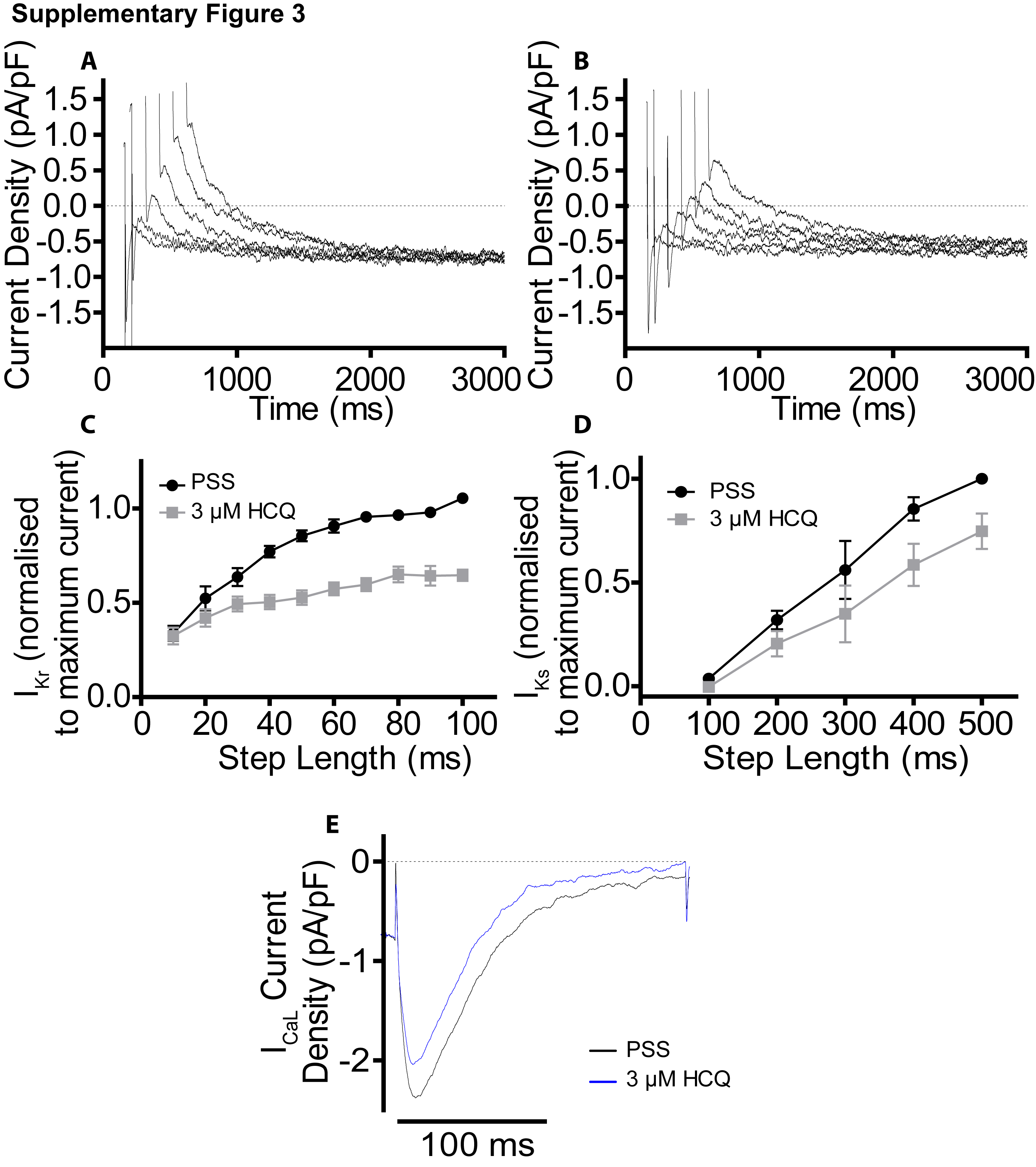

Supplement: Supplementary file 4 — Supplementary Material [file mmc4.tif]
